# Assessing Social Interaction and Loneliness and Their Association With Frailty Among Older Adults With Subjective Cognitive Decline or Mild Cognitive Impairment: Ecological Momentary Assessment Approach

**DOI:** 10.2196/64853

**Published:** 2025-04-22

**Authors:** Bada Kang, Dahye Hong, Seolah Yoon, Chaeeun Kang, Jennifer Ivy Kim

**Affiliations:** 1 Mo-Im Kim Nursing Research Institute Yonsei University College of Nursing Seoul Republic of Korea; 2 Institute for Innovation in Digital Healthcare Yonsei University Seoul Republic of Korea; 3 College of Nursing and Brain Korea 21 FOUR Project Yonsei University Seoul Republic of Korea; 4 Department of Nursing Yonsei University College of Nursing Seoul Republic of Korea

**Keywords:** geriatric, older, elderly, ageing, association, correlation, cognitive impairment, ecological momentary assessment, frailty, mild behavioral impairment, dementia, Alzheimer, isolation, lonely, social, interaction, self-reported, psychogeriatrics

## Abstract

**Background:**

Frail older adults are at greater risk of adverse health-related outcomes such as falls, disability, and mortality. Mild behavioral impairment (MBI), which is characterized by neurobehavioral symptoms in individuals without dementia, is a crucial factor in identifying at-risk groups and implementing early interventions for frail older adults. However, the specific role of social functioning, which encompasses social interaction and loneliness levels, in relation to frailty within this group remains unclear.

**Objective:**

This study investigated the association between frailty status, social interaction frequency, and loneliness levels among older adults with subjective cognitive decline (SCD) or mild cognitive impairment (MCI) while adjusting for MBI symptoms in 2 contexts: the presence and severity of MBI symptoms.

**Methods:**

Older adults with SCD or MCI were recruited from an outpatient clinic specializing in the early diagnosis and care management of dementia at a community health center, as well as from a community service center in Seoul, South Korea. Using an ecological momentary assessment approach, participants reported their daily social interaction frequency and loneliness level via a mobile app, 4 times daily for 2 weeks. Frailty status, the outcome variable, was assessed using the Korean version of the frailty phenotype questionnaire. Additionally, MBI symptoms were assessed using the 34-item MBI-Checklist covering 5 domains. Multinomial logistic regression analyses were performed to investigate the association between frailty status (robust, prefrail, and frail), and the independent variables, adjusting for the presence or severity of MBI symptoms.

**Results:**

Among the 101 participants analyzed, 29.7% (n=30) of participants were classified as prefrail, and 12.8% (n=13) of participants were classified as frail. Higher average daily social interaction scores were consistently associated with lower odds of a frail status compared to a robust status. This was evident in the models adjusted for both the global presence (relative risk ratio [RRR] 0.18, *P*=.02) and global severity (RRR 0.20, *P*=.02) of MBI symptoms.

**Conclusions:**

Frequent social interaction was inversely associated with frail status in older adults with SCD or MCI, even after adjusting for the presence and severity of MBI symptoms. These findings highlight the potential of social functioning as a modifiable factor for addressing frailty among older adults at risk of cognitive and functional decline. Future prospective studies using real-time measurements are needed to refine these findings and further investigate additional risk factors and functional outcomes in this group.

## Introduction

The demographic shift toward an increasingly aging population presents significant public health challenges, with a prominent focus on aging-related adverse health outcomes such as frailty. Frailty is a multidimensional syndrome characterized by a decline in physiological reserve across multiple systems, leading to increased vulnerability to stressors and adverse outcomes. These outcomes include physical limitations, such as impaired mobility, muscle weakness, fatigue, cognitive decline, social isolation, and psychological distress [1-3]. Frailty manifests through physical, cognitive, and psychological components, often resulting in increased risks of falls, disability, hospitalization, and mortality [1,2,4]. Physically, frailty impairs an individual’s ability to perform daily activities, while cognitively, it may exacerbate the risks of memory and attention deficits [1-6]. Social and psychological factors also play a role, as frailty is often associated with social isolation and depressive symptoms [3,7]. Frailty and prefrailty incidences were estimated at 43 and 151 new cases per 1000 person-years, respectively, indicating that approximately 1 in 6 community-dwelling older adults may be frail [8]. Frailty is more common in women, older individuals, those with a lower socioeconomic status, and individuals with comorbid chronic diseases and disabilities [1,8-10].

Subjective cognitive decline (SCD) refers to a self-perceived decline in cognitive performance without objective evidence of cognitive impairment [11,12]. Mild cognitive impairment (MCI) represents a state between normal cognition and dementia, characterized by a cognitive decline more pronounced than expected for an individual’s age and educational level, but without significant interference in daily activities [13,14]. The co-occurrence of frailty and early-stage cognitive decline has been well documented, with studies showing an increased likelihood of frailty in older adults with SCD or MCI. Two systematic reviews and meta-analyses have offered solid evidence of this relationship [15,16]. Specifically, a cross-sectional study of 2386 individuals with SCD revealed that they had a higher likelihood of being prefrail or frail, compared with individuals with normal cognition, even after controlling for factors such as sociodemographic characteristics, physical functionality, psychosocial aspects, and biochemical influences [12]. The Victoria Longitudinal Study, which followed 632 individuals without dementia, also showed that increased frailty was associated with a more rapid memory decline [5]. Therefore, it is essential to identify individuals with SCD or MCI who are at risk of becoming prefrail or frail, and to implement preventative interventions that focus on modifiable lifestyle and health factors.

Although not all cases of frailty and early cognitive impairment are reversible, evidence supports the potential reversibility of both conditions, including SCD and MCI, through the early detection of at-risk groups and the application of tailored interventions [2,15,17,18]. In addition, factors such as age, sex, and activities of daily living are associated with frailty and cognitive decline [19-22], and mild behavioral impairment (MBI) is also correlated with higher levels of frailty and an increased risk of cognitive decline [2,3]. MBI, which emerges later in life, is a neurobehavioral syndrome characterized by sustained and impactful neuropsychiatric symptoms across 5 domains: decreased drive and motivation, affective dysregulation, impulse dyscontrol, social inappropriateness, and abnormal perception or thought content [23]. MBI is considered a precursor to, or concurrent with, MCI, affecting older adults with either normal cognition or SCD [23]. Significant associations have been observed between certain MBI domains and increased frailty risk in individuals with MCI [3]. Additionally, frailty was associated with both the presence and severity of MBI, especially in men [2]. These findings underscore the importance of assessing MBI to detect risks before the onset of frailty and dementia and warrant the application of interventions for its potential modification.

Psychosocial functioning, which encompasses how individuals engage with society and adapt to environmental challenges, is also an influential factor associated with frailty [24]. Infrequent social interactions, which reflect the quantitative aspects of an individual’s limited contact with others [7], and loneliness or perceived social isolation are important factors in this context [25]. Although a definitive theory connecting limited social interaction, loneliness, and frailty has yet to be established, various studies have indicated that reducing social isolation and loneliness in later life can significantly improve overall well-being, underscoring the need for collective consideration of how these factors impact physical health [7]. The English Longitudinal Study of Aging demonstrated that social isolation and loneliness increased the risk of frailty in older adults [26]. Furthermore, given the significant impact of social isolation and loneliness, addressing even moderate levels of these factors is important, as they are associated with an increased risk of worsening frailty [27]. Understanding these psychosocial factors is vital in the broader aging context, as they affect not only physical health but also psychological well-being, thereby potentially influencing older adults’ overall quality of life and health care needs [7]. Therefore, the interplay between psychosocial factors and health outcomes underscores the importance of holistic approaches in developing geriatric and preventive strategies.

The ecological momentary assessment (EMA) provides a dynamic and immediate record of personal experiences. It is particularly effective for older adults with cognitive decline, offers a comprehensive view of their everyday social lives, and yields more nuanced insights than conventional global measures [28]. By capturing patterns of daily social interaction or loneliness levels, the EMA addresses restrictions on time or recall bias [29]. However, the relationship between frailty, social interaction, and loneliness levels, particularly in the context of MBI symptomatology, remains underexplored, while few studies have used EMA to examine the real-time social functional status among older adults with cognitive decline. Therefore, this study aimed to investigate the cross-sectional relationships between social interaction frequency, loneliness levels, and frailty status among older adults with SCD or MCI in two contexts: (1) adjusting for the presence of MBI symptoms and (2) adjusting for the severity of MBI symptoms. We hypothesized that, similar to MBI symptoms, both the frequency of social interactions and the level of loneliness would be significantly correlated with frailty. This exploration is anticipated to yield deeper insights into psychosocial factors related to frailty among individuals with SCD or MCI.

## Methods

### Design

**Figure 1:**
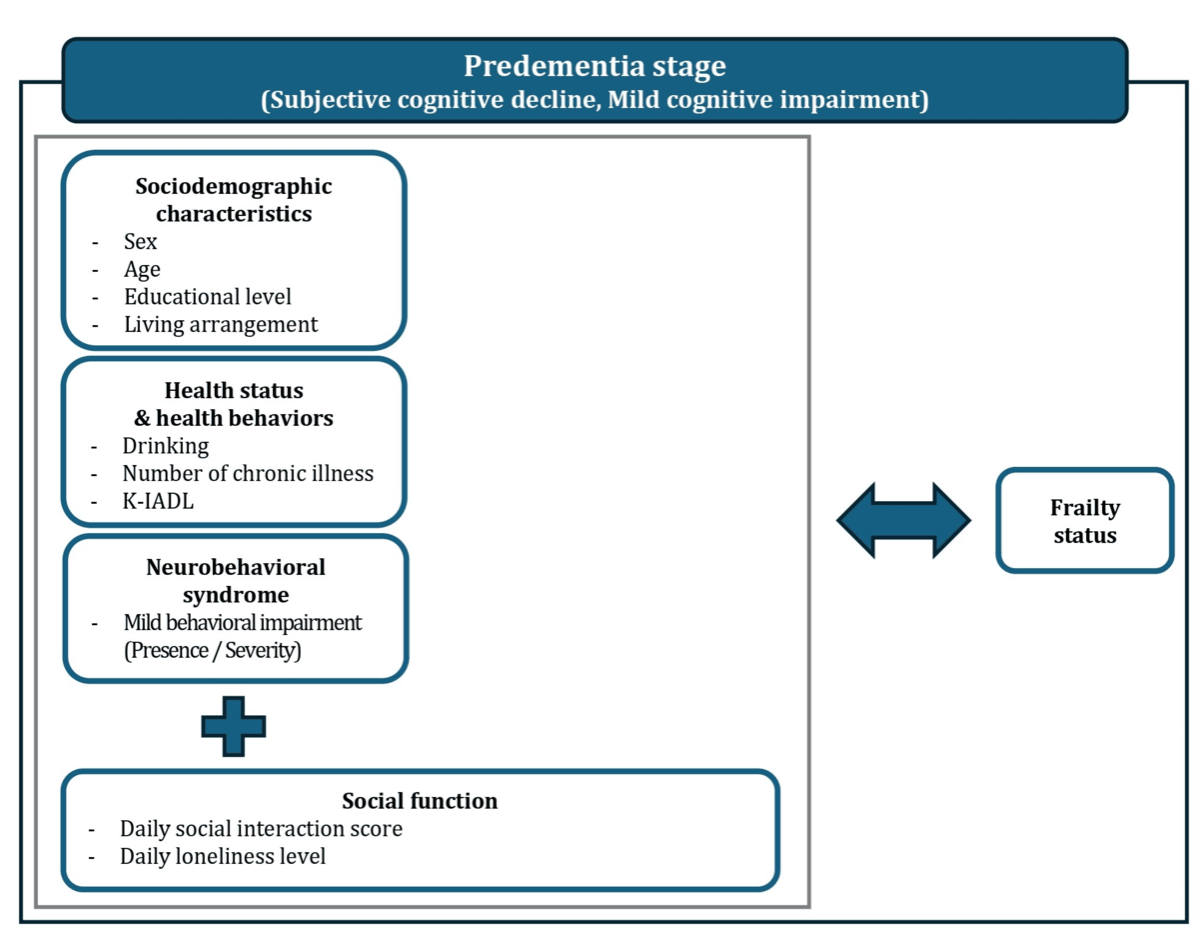
The proposed conceptual framework illustrates the relationship between frailty status, social interaction, and loneliness levels, while adjusting for the presence and severity of mild behavioral impairment symptoms among older adults with subjective cognitive decline or mild cognitive impairment. Based on the social-ecological model for older adults and the successful aging model proposed by Rowe and Kahn, individual determinants include sociodemographic characteristics, health status, and health behaviors. Social function, including daily social interaction frequency and daily loneliness level, is also considered. K-IADL: Korean Instrumental Activities of Daily Living scale.

Our study represents the first wave of a 3-year prospective longitudinal study aimed at developing functional prediction models based on behavioral and psychosocial indicators for older adults with SCD or MCI. The study protocol was published elsewhere [30]. The following is a detailed explanation of the variables considered in this study. We constructed the conceptual framework of our study, based on the social-ecological model for older adults [31] and the successful aging model proposed by Rowe and Kahn [32], as shown in Figure 1.

### Participants and Setting

For the larger 3-year longitudinal study, we initially estimated the required sample size using R (generalized linear mixed model, effect size=0.30, k=2, power=0.80, α=0.05, df=30), yielding a minimum of 106 participants. The study protocol has been published elsewhere [30]. The effect size was selected based on prior studies examining the relationship between frailty and social-behavioral factors, which reported moderate effect sizes in similar aging populations [33,34]. Based on these findings, we adopted an effect size of 0.30 as a conservative estimate, to reflect a moderate expected effect while ensuring robustness in our calculation. To account for potential attrition, we adjusted the sample size to 127 participants, anticipating a 20% dropout rate. This adjustment aligns with established guidelines that emphasize careful assessment of attrition rates in aging populations, particularly given the health-related challenges that contribute to higher dropout rates [35,36].

We initially recruited 145 individuals from a dementia relief center and a community service center in Seoul, South Korea (SCD: n=91; MCI: n=54) between October 2022 and November 2023. The dementia relief center houses an outpatient clinic that specializes in early diagnosis and care management of dementia, whereas the community service center serves as a place for older adults to engage in recreational and wellness programs, irrespective of a dementia diagnosis. Specific criteria were applied to recruit study participants from each center. The common inclusion criteria for the study participants were as follows: (1) older than 65 years, (2) ability to use a smartphone, (3) ability to respond to momentary questionnaires via a mobile app, and (4) ability to provide written consent for participation.

Individuals who met the inclusion criteria were selected for the SCD group. Participants were either (1) individuals from the dementia relief center who, according to a nurse’s report, had neither been diagnosed with MCI nor dementia; or (2) individuals recruited from the community service center through convenience sampling, who self-reported having no history of MCI or dementia diagnosis and scored above 24 on the Korean Mini-Mental State Examination, second edition. Further, we confirmed SCD in individuals from both the dementia relief center and the community service center by ensuring that they provided an affirmative response to the question, “Do you think your memory has gotten worse, compared with the previous year?”

The MCI group consisted exclusively of participants clinically diagnosed with MCI by medical doctors at the dementia relief center. This group was screened to include only individuals who scored above 18 on the Korean Mini-Mental State Examination, second edition. Participants were excluded if they were (1) illiterate; (2) diagnosed with neurological diseases such as epilepsy, stroke, Parkinson disease, or other types of brain damage; (3) diagnosed with psychiatric diseases such as schizophrenia or bipolar disorder; and (4) undergoing critical illness treatments such as chemotherapy, had severe cardiovascular disease, or had abused substances (including narcotics or alcohol) within the last 3 years.

**Figure 2:**
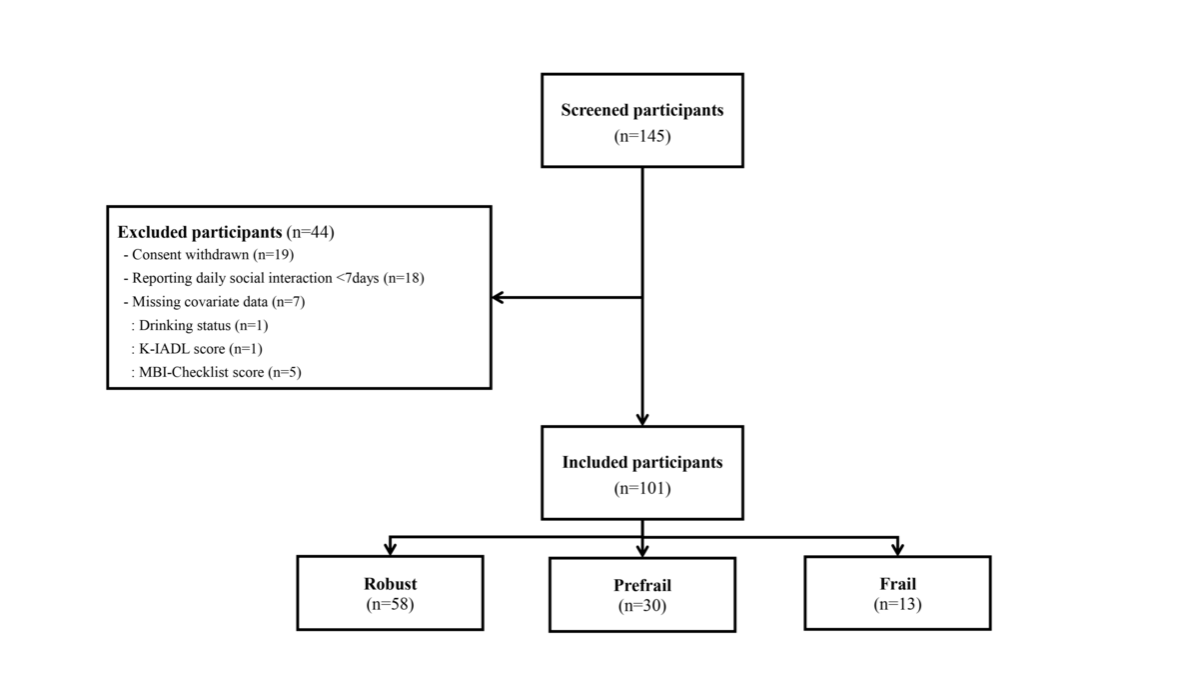
Sequential steps involved in the recruitment and selection of study participants. We recruited 145 individuals aged ≥65 years from a dementia relief center or a community service center in Seoul, South Korea. The figure depicts the subdivision of these individuals into 3 groups: 58 with robust, 30 with prefrail, and 13 frail, based on specific inclusion and exclusion criteria. K-IADL: Korean Instrumental Activities of Daily Living scale; MBI: mild behavioral impairment.

After excluding individuals who withdrew their consent (n=19), those who reported EMA responses for less than a week or reported no EMA responses per day (n=18), and those with missing covariate data (n=7), 101 individuals were included in the final sample. The participant selection process is illustrated in Figure 2.

### Measures

#### Frailty

Frailty status was the primary outcome variable in this study. Frailty was assessed using the Korean version of the frailty phenotype questionnaire (FPQ), which is specifically designed to screen for the Fried Frailty Phenotype in community-dwelling older adults [37]. While the Fried Frailty Phenotype requires direct measurements of gait speed, grip strength, and physical activity, the FPQ has been validated as an effective proxy for these assessments. Among Korean older adults, the FPQ demonstrated satisfactory diagnostic accuracy for the Fried Frailty Phenotype, with an area under the curve of 0.89, a high sensitivity of 81.7%, and a specificity of 82.5% [37].

The FPQ is particularly well-suited for epidemiological research due to its low resource requirements and ease of administration, making it a practical screening tool for community-dwelling older adults where detailed clinical assessments may not be feasible. The FPQ comprises 5 questions addressing the key aspects of frailty: fatigue (exhaustion), resistance (weakness), ambulation (slowness), inactivity, and weight loss. Scoring from 0 to 5, where 0 indicates robustness, 1-2 indicates a prefrail status, and ≥ 3 indicates a frail status.

#### Daily Social Interaction Frequency and Level of Loneliness

Daily social interaction frequency and level of loneliness were the main independent variables. Daily social interaction frequency was measured to reflect quantifiable aspects of social contact, while the level of loneliness was assessed to capture one’s perceived view of the consequences of social contact [7]. While the level of loneliness is often measured with a single directional question about one’s loneliness status, a solid assessment method for social interaction has yet to be established [7]. Despite the complexity and heterogeneity of its indicators, social isolation can be quantitatively assessed by aggregating various factors into a composite index [7]. Building on a prior study that determined social activity frequency by inquiring about the frequency of participant engagement in socially interactive activities among older adults without clinical signs of dementia rated on a 5-point scale [38], this study quantitatively assessed the daily frequency of social interaction.

Given the cognitive challenges faced by our participants with SCD or MCI, simplifying the data collection process was essential to reduce participant burden and ensure consistent and reliable responses. For this reason, we did not differentiate between types of social interactions (eg, in-person, phone-based), as the study’s primary goal was to explore the overall relationship between social engagement and frailty in older adults with cognitive decline.

The EMA method was particularly well-suited for this purpose because it allowed us to collect real-time data on both the frequency of social interactions and participants’ perceived loneliness. This approach minimizes recall bias, particularly relevant for individuals with cognitive difficulties. A mobile EMA app was used. With smartphone ownership rates among older Korean adults in 2023 reaching 97.2% for those aged 60-69 years and 72.9% for those aged 70 years and older [39], the feasibility of this method was significantly enhanced.

**Figure 3:**
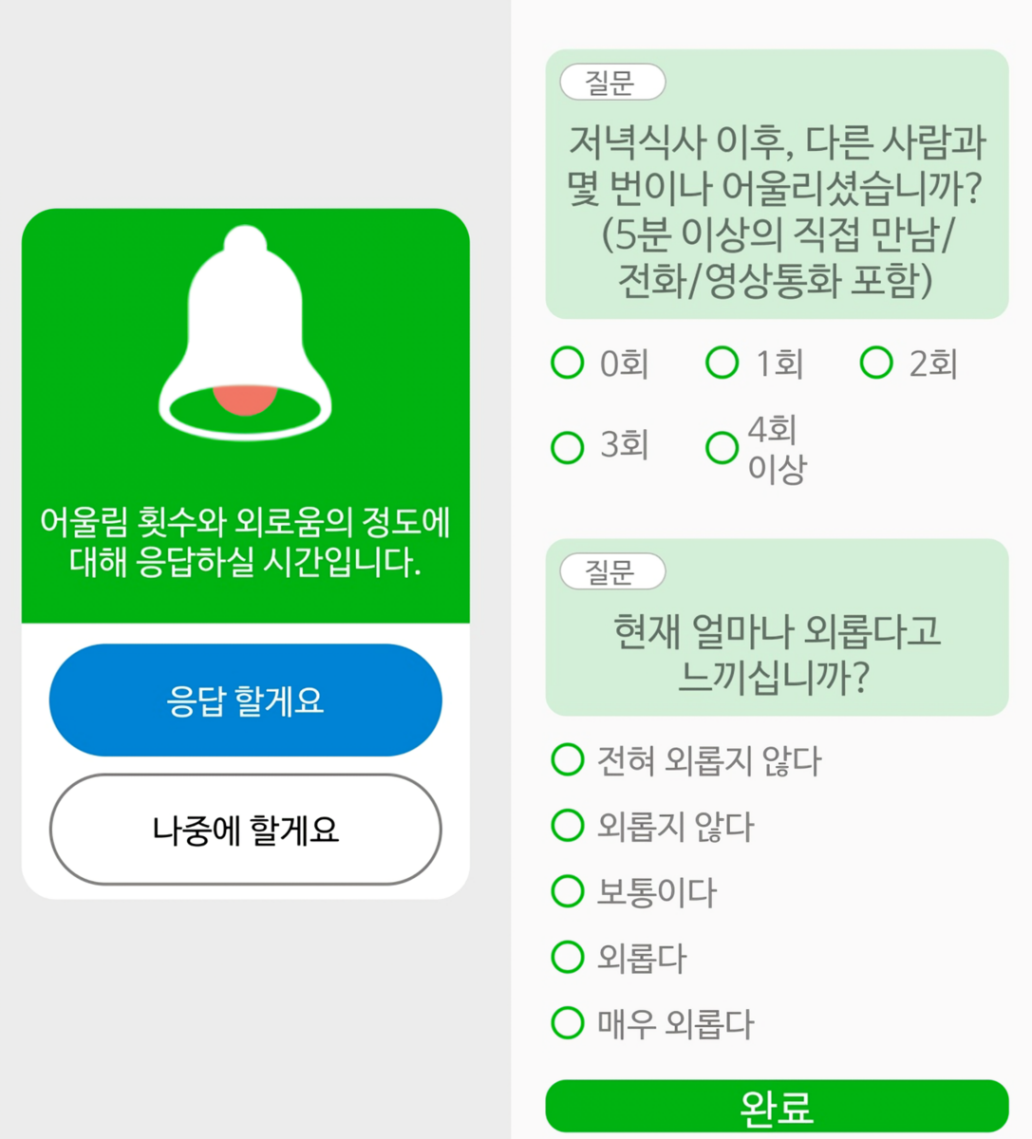
The mobile app screens captured 2 questions that assessed daily social interaction frequency and loneliness levels. Daily social interaction frequency was measured by asking, “How many times did you interact with others, including face-to-face meetings, phone calls, or video calls lasting more than five minutes?” Responses were categorized into 5 options: no contact, once, twice, 3 times, and 4 or more times. Loneliness levels were measured by asking, “At this moment, how lonely do you feel?” Responses were also categorized into 5 options: very lonely, lonely, neutral, not lonely, and not lonely at all. Both questions were administered 4 times daily at specific intervals (upon waking and after breakfast, lunch, and dinner).

The EMA method facilitated real-time data collection through a mobile phone app that set off alarms 4 times daily (upon waking and 3 times during mealtimes) over 2 weeks. Each alarm prompted participants to answer 2 questions. The first question assessed the frequency of social interactions since upon waking or since the last mealtime: “How many times have you had a social encounter?” Participants recorded the number of face-to-face meetings, phone calls, or video calls lasting more than 5 minutes using a 5-point Likert scale (0 for “no contact,” 1 for “once,” 2 for “two times,” 3 for “three times,” and 4 for “four or more times”). The second question assessed loneliness: “At this moment, how lonely do you feel?” using a similar 5-point Likert scale (0=“very lonely” to 4=“not lonely at all”). Both questions were asked 4 times a day at set intervals (upon waking and after breakfast, lunch, and dinner). Figure 3 shows the captured screens from the mobile app. The research staff provided follow-up contacts for troubleshooting as needed. Participants received gifts worth US $50 after completing the 2-week data collection period.

We implemented 4 strategies to ensure accurate EMA data collection. First, we provided comprehensive training sessions, including repeated practice, to ensure participants could use the mobile app independently. Second, we provided detailed instructional manuals for completing the response process (Multimedia Appendices 1-3). Although the EMA questions are simple, we addressed potential challenges given that the participants were individuals with SCD or MCI. Third, we established a helpdesk for assistance with any app-related issues during the follow-up period. Last, we offered direct support as needed, with written contact information provided to ensure participants could reach researchers via phone or in person. These measures aimed to enhance participants’ understanding and proficiency, contributing to more accurate data collection. Approximately 15 participants requested assistance during the study, mainly due to issues with mobile EMA alarms not triggering due to internet connectivity issues or difficulties with smartphone lock functions. Researchers provided phone guidance and, when necessary, conducted in-person visits to resolve the issues and offered additional training.

The quantity of social interaction and the level of loneliness were indicated as daily mean scores based on previous studies [28,40,41]. Data processing involved calculating the maximum daily social interaction frequency as the final daily score. For instance, if a participant reported varying frequencies of social encounters throughout day 1, the highest frequency reported that day was recorded as the final score. The average daily social interaction score was then determined by summing the scores and dividing them by the total number of valid responses. Similarly, the average daily loneliness score was calculated by adding the total loneliness scores recorded each day (with at least one response) and dividing them by the total number of valid response days.

#### Presence and Severity of MBI Symptoms

The presence and severity of MBI symptoms were included as key covariates. The Mild Behavioral Impairment Checklist (MBI-C) was used to assess the onset of sustained and impactful neurocognitive symptoms in the predementia stage [23]. The MBI-C systematically evaluates symptoms across the five domains of MBI, persisting for at least 6 months: (1) decreased motivation, (2) affective dysregulation, (3) impulse dyscontrol, (4) social inappropriateness, and (5) abnormal perception or thought content. Each symptom was initially assessed dichotomously (yes/no), followed by a severity rating of mild (1), moderate (2), or severe (3) [23].

In this study, the Korean version of the MBI-C was used to evaluate the presence and severity of MBI symptoms. The MBI-C, a brief screening tool previously validated, was implemented to detect MBI in line with established criteria [3]. The MBI-C has been translated into various languages including Korean. Psychometric evaluation of the Korean version demonstrated significant correlations with the Neuropsychiatric Inventory in individuals with amnestic MCI (*r*=0.25, *P*<.01) and non-amnestic MCI (*r*=0.36, *P*<.05) [42,43]. In this study, Cronbach α for the MBI-C was 0.89. For presence assessment, we used a dichotomous method for both domain-specific and global MBI symptoms, with a cut-off score > 0 indicating the presence of MBI symptoms [2]. Severity was assessed as either (1) domain-specific, where the sum of scores from each individual domain was calculated; or (2) global, involving the cumulative sum of scores across all domains [2].

#### Covariates

Frailty encompasses numerous biological, physiological, and environmental changes that typically occur during the aging process [21]. Therefore, we considered sociodemographic and health-related characteristics, health behaviors, and functional limitations as covariates. The demographic characteristics of participants included sex (male/female), age group (<70, 70-74, 75-79, and ≥80 years), educational level (<9/≥9 years), and living arrangement (living alone/residing with others).

For health-related characteristics, we considered the total number of chronic illnesses diagnosed, including hypertension, diabetes, cardiovascular diseases, cerebrovascular diseases, gastrointestinal diseases, chronic respiratory diseases, chronic kidney diseases, and musculoskeletal conditions. Participants’ cognitive status was dichotomously categorized as SCD or MCI based on their responses, which were recorded to evaluate the inclusion criteria. Health behavior aspects included drinking status (active drinker/nondrinker). Owing to the limited sample size of former or current smokers (n=2), smoking status was excluded from the statistical analysis.

As demonstrated in previous studies, dependence on managing higher levels of functional performance is associated with frailty [21,22]. Therefore, functional limitations were assessed using the Korean Instrumental Activities of Daily Living (K-IADL) scale, which measures the ability to perform complex daily tasks such as cooking, grooming, housekeeping, shopping, financial management, transportation, laundry, short-distance walking, making phone calls, and managing medications [44]. Each item on the K-IADL scale is rated from 0 to 4, with higher scores indicating greater dependency. The K-IADL’s validation, conducted with older adults aged older than 65 years, demonstrated strong internal consistency (Cronbach α=0.94), high interrater reliability (κ value=0.81-0.95), and test-retest reliability correlation (≥ 0.70) [44]. In this study, high K-IADL scores indicated diminished functional capabilities and were analyzed as continuous variables.

#### Statistical Analysis

Descriptive statistics were used to outline the general characteristics of the participants and compare differences between the robust, prefrail, and frail groups. Categorical variables are presented as counts and percentages, while continuous variables are summarized as means with SDs.

To assess the normality of continuous variables, we performed the Shapiro-Wilk test. If the data were normally distributed and variance was equal, analysis of variance was applied to compare differences between groups. When normality was not met or variance was unequal, the Kruskal-Wallis test was used. A chi-square test was used to assess differences in categorical variables between groups, with the Fisher exact test applied when the expected cell count was less than 5.

We conducted multinomial logistic regression analyses to examine the association between frailty status, social interaction frequency, and loneliness levels among older adults with MCI or SCD. Frailty status was the primary outcome variable and was categorized into 3 groups: robust, prefrail, and frail. Social interaction frequency and daily loneliness levels were the independent variables, assessed using EMA.

To account for potential confounding effects, MBI symptoms were included as a key covariate. Additionally, other confounders, including sex, age group, educational level, living arrangement, number of chronic illnesses, cognitive status, health behaviors, and functional limitations, were selected based on their established relevance to frailty and social engagement in previous studies [2,3,19-22].

Separate multinomial logistic regression models were performed to adjust for MBI symptom presence and severity. All independent variables were simultaneously entered into the model to ensure consistency and comparability across analyses. Multicollinearity was assessed using the variance inflation factor to confirm that the included variables were not highly correlated. Model fit was evaluated using the generalized Hosmer-Lemeshow test and model specification was checked for validity. Statistical significance was set at *P*<.05 and all analyses were performed using Stata software (version 16; StataCorp).

### Ethical Considerations

This study was approved by the Institutional Review Board of Severance Hospital, Yonsei University Health System (number 4-2022-0637). In accordance with ethical guidelines, all participants provided their written informed consent prior to their inclusion in the study. To ensure ethical integrity, individuals with severe cognitive decline who were assessed as incapable of making informed decisions regarding participation were excluded from the study.

## Results

### Participants’ General Characteristics

Table 1 presents the general characteristics of participants. A total of 101 participants were included in the analysis; 58 (57.4%) were categorized as robust, 30 (29.7%) as prefrail, and 13 (12.9%) as frail. Significant differences were observed between the groups in age and global MBI symptom severity. The mean age and SDs for each group were robust (74.9, SD 5.1 years), prefrail (75.7, SD 6.5 years), and frail (82.0, SD 4.9 years), with corresponding age ranges of 65-87, 65-89, and 73-89 years, respectively. For global MBI severity, the median score for all participants was 3 (IQR 1-9.5). The robust group had the lowest median severity score (2, IQR 1-4.5), while the frail group exhibited the highest severity (9, IQR 3-23.5).

**Table 1:** Baseline characteristics of participants by frailty status.

Variables			Total (N=101)		Robust (n=58)		Prefrail (n=30)		Frail (n=13)		*P* value	
**Sex, n (%)**												.47
	Men	42 (41.6)		27 (46.5)		10 (33.3)		5 (38.5)				
	Women	59 (58.4)		31 (53.5)		20 (66.7)		8 (61.5)				
**Age (years)**												
	Range	65-89		65-87		65-89		73-89		—^a^		
	Mean (SD)	76.1 (5.9)		74.9 (5.1)		75.7 (6.5)		82.0 (4.9)		<.001		
**Educational level** **, n (%)**												.06
	<9 years	30 (29.7)		12 (20.7)		12 (40.0)		6 (46.2)				
	≥9 years	71 (70.3)		46 (79.3)		18 (60.0)		7 (53.8)				
**Living arrangement^b^, n (%)**												.22
	Living alone	19 (18.8)		8 (13.8)		9 (30.0)		2 (15.4)				
	Residing with others	82 (81.2)		50 (86.2)		21 (70.0)		11 (84.6)				
**Cognitive status, n (%)**												.06
	SCD^c^	67 (66.3)		44 (75.9)		16 (53.3)		7 (53.9)				
	MCI^d^	34 (33.7)		14 (24.1)		14 (46.7)		6 (46.1)				
**Drinking status^b^, n (%)**												.46
	Active drinker	25 (24.8)		17 (29.3)		5 (16.7)		3 (23.1)				
	Nondrinker	76 (75.2)		41 (70.7)		25 (83.3)		10 (76.9)				
Number of chronic illnesses, mean (SD)			2.01 (1.11)		1.96 (1.07)		1.93 (1.14)		2.46 (1.19)		.31	
**K-IADL^e,f^**												
	Range	1-17		1-16		8-17		9-16		—		
	Median (IQR)	10 (10-10)		10 (10-10)		10 (10-10)		10 (10-12.5)		—		
	Mean (SD)	10.4 (1.9)		10.4 (2.1)		10.4 (1.7)		11.0 (1.9)		.64		
Global MBI^g^ symptom presence, n (%)			81 (80.2)		45 (77.5)		24 (80.0)		12 (92.3)		.64	
**Global MBI^g^ symptom severity^f^**												
	Range	0-41		0-25		0-40		0-41		—		
	Median (IQR)	3 (1-9.5)		2 (1-4.5)		8.5 (1-12.3)		9 (3-23.5)		—		
	Mean (SD)	6.9 (9.0)		4.0 (5.1)		9.2 (10.1)		14.5 (13.8)		.002		

^a^Not applicable.

^b^Fisher exact test was applied.

^c^SCD: subjective cognitive decline.

^d^MCI: mild cognitive impairment

^e^K-IADL: Korean Instrumental Activities of Daily Living scale.

^f^Kruskal-Wallis test was applied.

^g^MBI: mild behavioral impairment.

Table 2 displays a descriptive summary of social interaction frequency and loneliness levels for all participants, stratified by frailty status. We summarized the average maximum daily social interaction score by calculating the average of each participant’s daily maximum scores across the study period, resulting in a single, average score per participant. No significant differences were observed in the mean average maximum daily social interaction scores among the groups: robust (3.0, SD 0.8), prefrail (2.9, SD 0.9), and frail (2.4, SD 0.9). Similarly, no significant differences were found in the mean average maximum daily loneliness levels, with scores of 3.4 (SD 0.5) for the robust group, 3.3 (SD 0.6) for the prefrail group, and 3.1 (SD 0.9) for the frail group.

**Table 2:** Descriptive statistics of daily social interaction frequency and loneliness levels by frailty status.

		Total (N=101)	Robust (n=58)	Prefrail (n=30)	Frail (n=13)	*P* value
**Social interaction frequency per day**						
	Range	0-4	0-4	0-4	0-4	
	Mean (SD)	2.9 (0.8)	3.0 (0.8)	2.9 (0.9)	2.4 (0.9)	.08
**Loneliness level per day**						
	Range	0-4	1-4	0-4	0-4	
	Mean (SD)	3.4 (0.6)	3.6 (0.5)	3.3 (0.6)	3.1 (0.9)	.06

**Figure 4:**
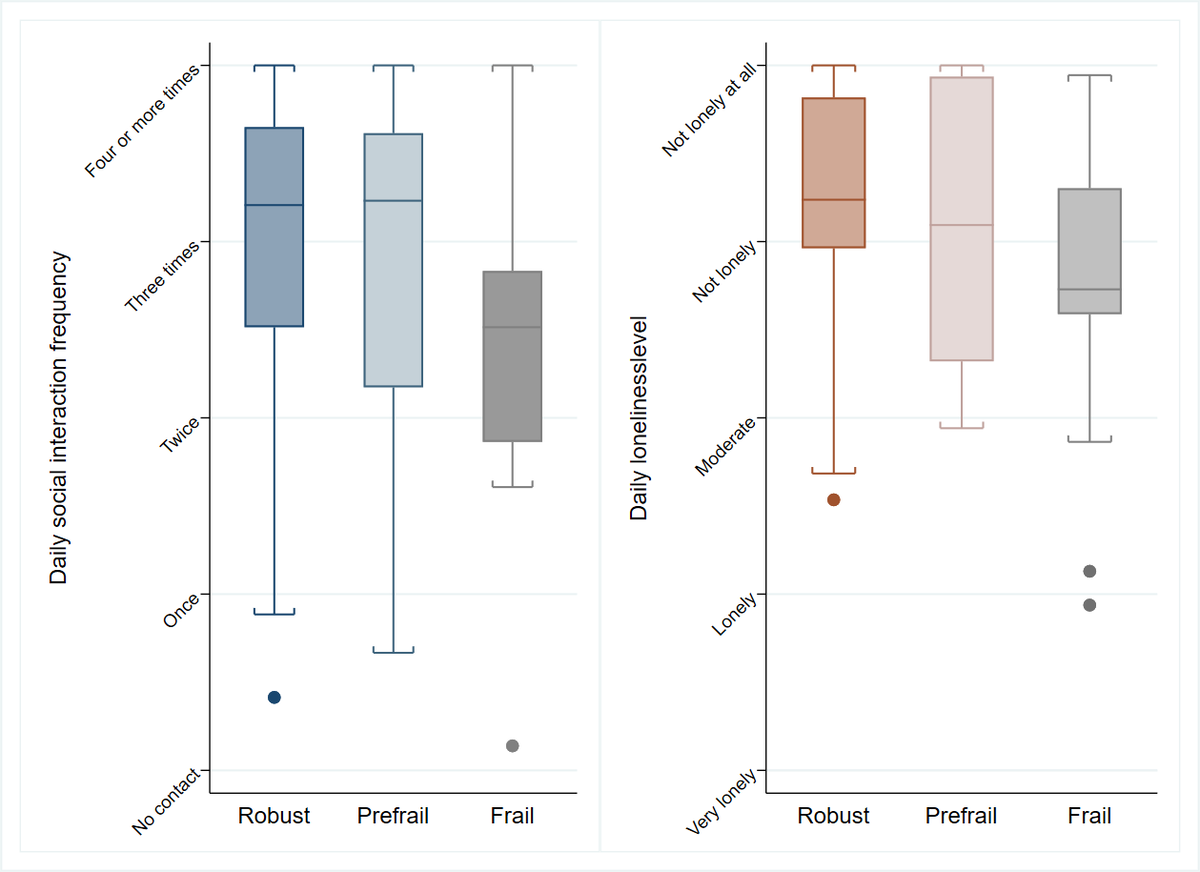
Distribution of average daily social interaction score and average daily loneliness level by frailty status. This figure displays the median levels and range variability of both average daily social interaction score and average daily loneliness levels among participants, categorized into robust, prefrail, and frail groups.

Figure 4 illustrates the variations of the average daily social interaction scores and average daily loneliness levels categorized by frailty status. The robust group is characterized by the highest median values in daily social interactions and the lowest loneliness levels compared with prefrail and frail groups. In contrast, the frail group demonstrates the lowest median social interaction frequency and the highest loneliness levels. Furthermore, the density distribution of mean maximum daily social interaction scores and mean maximum daily loneliness level by frailty status is illustrated in Multimedia Appendices 4 and 5. While the overall distribution is right skewed across all participants, the frail group exhibits a relatively higher density in the lower frequency of social interactions compared with the robust or prefrail group (Multimedia Appendix 4). In addition, the frail group shows a relatively lower density of participants experiencing lower loneliness levels compared with robust and prefrail groups, even though the overall distribution is right-skewed (Multimedia Appendix 5).

### Primary Outcomes

#### Association Between Daily Social Interaction Frequency, Daily Loneliness Level, and Frailty Adjusting for the Presence of Global MBI Symptoms

**Figure 5:**
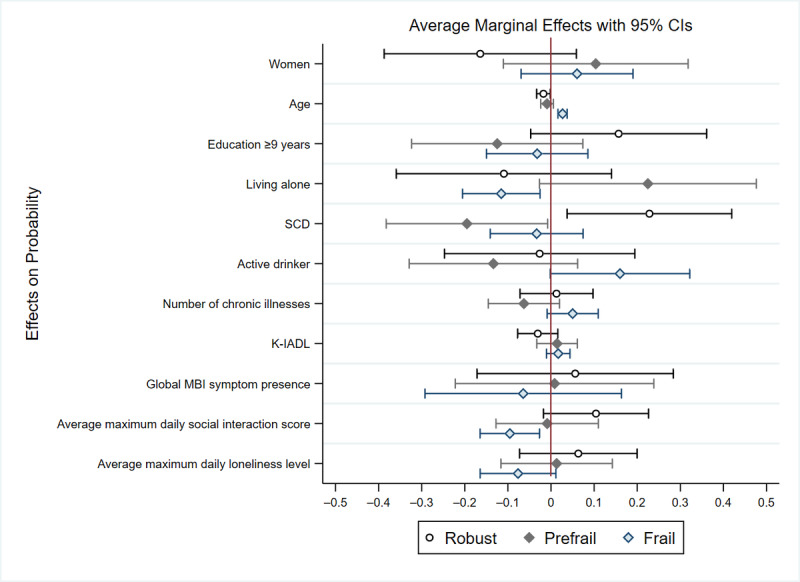
This figure shows the average predictive marginal effects of sociodemographic characteristics, health status, global MBI symptom presence, and social functioning on the probability of being classified as robust, prefrail, or frail. The predictive margins were calculated using multinomial logistic regression, with error bars representing 95% CIs for each effect. K-IADL: Korean Instrumental Activities of Daily Living scale; MBI: mild behavioral impairment; SCD: subjective cognitive decline.

Table 3 illustrates the relationship between frailty, daily social interaction frequency, and daily loneliness level after adjusting for the presence of global MBI symptoms. After adjusting for the presence of global MBI symptoms, neither the average maximum daily social interaction score (relative risk ratio [RRR] 0.74, *P*=.42) nor the average maximum daily loneliness level (RRR 0.89, *P*=.76) were significantly associated with prefrail status. Similarly, no significant relationship was observed between the average maximum daily loneliness level and frail status (RRR 0.27, *P*=.10). However, participants with higher average maximum daily social interaction scores were significantly less likely to be frail (RRR 0.18, *P*=.02). Sociodemographic and health-related characteristics also showed significant associations with frailty. Participants with SCD were less likely to be prefrail compared with those with MCI (RRR 0.31, *P*=.03), while older age was associated with a higher likelihood of being frail (RRR 1.56, *P*=.001). Figure 5 displays the average predictive marginal effects of sociodemographic characteristics, health status, global MBI symptom presence, and social functioning on the probability of being classified as robust, prefrail, or frail.

**Table 3:** Multinomial logistic regression model for the association between frailty, global MBI-symptom presence, daily social interaction frequency, and level of loneliness (N=101) (Wald χ222=52.04).

Variables	Prefrail (base outcome: robust)			Frail (base outcome: robust)	
	RRR^a^ (95% CI)	*P* value	RRR (95% CI)		*P* value
Women (Ref: men)	2.18 (0.57-8.26)	.25	3.54 (0.42-29.72)		.24
Age (years)	1.01 (0.92-1.12)	.79	1.56 (1.20-2.01)		.001
Educational level ≥ 9 years (Ref: <9 years)	0.46 (0.16-1.33)	.15	0.43 (0.06-2.89)		.38
Living alone (Ref: Residing with others)	2.44 (0.68-8.68)	.17	0.11 (0.003-3.27)		.20
SCD^b^ (Ref: MCI^c^)	0.31 (0.11-0.87)	.03	0.35 (0.06-2.17)		.26
Active drinker (Ref: Nondrinker)	0.62 (0.15-2.51)	.50	9.10 (0.61-135.11)		.11
Number of chronic illnesses	0.78 (0.48-1.26)	.31	2.04 (0.73-5.66)		.17
K-IADL^d^	1.13 (0.86-1.49)	.37	1.38 (0.85-2.25)		.18
Global MBI^e^-symptom presence	0.89 (0.25-3.15)	.85	0.36 (0.01-8.91)		.53
Average maximum daily social interaction score	0.74 (0.36-1.53)	.42	0.18 (0.05-0.72)		.02
Average maximum daily loneliness level	0.89 (0.41-1.92)	.76	0.27 (0.05-1.31)		.10

^a^RRR: relative risk ratio.

^b^SCD: subjective cognitive decline.

^c^MCI: mild cognitive impairment.

^d^K-IADL: Korean Instrumental Activities of Daily Living scale.

^e^MBI: mild behavioral impairment.

#### Association Between Daily Social Interaction Frequency, Daily Loneliness Level, and Frailty Adjusting for the Severity of Global MBI Symptoms

**Figure 6:**
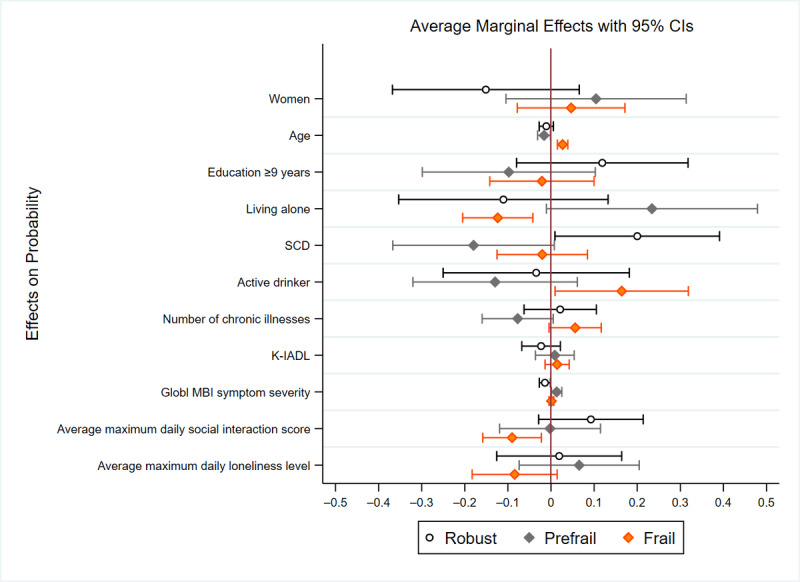
This figure shows the average predictive marginal effects of sociodemographic characteristics, health status, global MBI symptom severity, and social functioning on the probability of being classified as robust, prefrail, or frail. The predictive margins were calculated using multinomial logistic regression, with error bars representing 95% CIs for each effect. K-IADL: Korean Instrumental Activities of Daily Living scale; MBI: mild behavioral impairment; SCD: subjective cognitive decline.

Table 4 presents the associations between frailty, daily social interaction frequency, and daily loneliness levels after adjusting for the severity of global MBI symptoms. When adjusting for the severity of global MBI symptoms, no significant associations were observed between prefrail status and either the average maximum daily social interaction score (RRR 0.76, *P*=.48) or the average maximum daily loneliness level (RRR 1.19, *P*=.69). Likewise, no significant relationship was identified between frail status and average maximum daily loneliness level (RRR 0.27, *P*=.14). However, a significant inverse relationship was found between the average maximum daily social interaction scores and frail status (RRR 0.20, *P*=.02). Additional sociodemographic and health-related factors were associated with prefrail status. Participants with SCD were less likely to be prefrail compared with those with MCI (RRR 0.33, *P*=.04). Moreover, participants with more severe MBI symptoms were more likely to be prefrail (RRR 1.09, *P*=.04), and older age remained significantly associated with being frail (RRR 1.53, *P*=.002). Figure 6 presents the average predictive marginal effects of sociodemographic characteristics, health status, global MBI symptom severity, and social functioning on the probability of being classified as robust, prefrail, or frail.

**Table 4:** Regression models for the association between frailty, global MBIa-symptom severity, daily social interaction frequency, and level of loneliness (N=101; Wald χ222=56.59).

Variables	Prefrail (Base outcome: Robust)			Frail (Base outcome: Robust)	
	RRR^b^ (95% CI)	*P* value	RRR (95% CI)		*P* value
Women (Ref: men)	2.22 (0.56-8.71)	.25	2.93 (0.38-22.34)		.30
Age (years)	0.97 (0.88-1.10)	.63	1.53 (1.16-2.02)		.002
Educational level ≥9 years (Ref: <9 years)	0.52 (0.17-1.59)	.26	0.54 (0.08-3.65)		.52
Living alone (Ref: Residing with others)	2.58 (0.69-9.69)	.16	0.08 (0.002-2.57)		.16
SCD^c^ (Ref: MCI^d^)	0.33 (0.11-0.97)	.04	0.44 (0.07-2.57)		.36
Active drinker (Ref: Nondrinker)	0.65 (0.15-2.68)	.55	10.29 (0.61-174.13)		.11
Number of chronic illnesses	0.72 (0.44-1.18)	.20	2.14 (0.74-6.12)		.15
K-IADL^e^	1.10 (0.84-1.44)	.49	1.31 (0.81-2.13)		.27
Global MBI^f^-symptom severity	1.09 (1.01-1.18)	.04	1.06 (0.95-1.17)		.32
Average maximum daily social interaction score	0.76 (0.36-1.61)	.48	0.20 (0.05-0.78)		.02
Average maximum daily loneliness level	1.19 (0.51-2.78)	.69	0.27 (0.05-1.56)		.14

^a^MBI: mild behavioral impairment.

^b^RRR: relative risk ratio.

^c^SCD: subjective cognitive decline.

^d^MCI: mild cognitive impairment.

^e^K-IADL: Korean Instrumental Activities of Daily Living scale.

^f^MBI: mild behavioral impairment.

## Discussion

### Principal Findings

This study demonstrated a significant association between frailty and the frequency of daily social interaction in a cohort of 101 individuals at risk of dementia in South Korea. Specifically, higher average daily social interaction scores were significantly associated with lower relative risk ratios of being frail, even after adjusting for the presence and severity of global MBI symptoms. These findings underscore the potential role of daily social interaction frequency as a modifiable behavioral factor in individuals at the early stages of dementia and frailty risk.

Our study found that frail older adults exhibited more severe MBI symptoms than robust or prefrail groups. This observation aligns with prior research suggesting a potential link between frailty and the severity of MBI symptoms [2]. Furthermore, our findings indicate that frail older adults had lower average maximum daily social interaction frequency and higher loneliness levels compared with robust or prefrail groups. Unlike previous studies that assessed social interaction or loneliness at a single time point [7], our study used a 2-week tracking period. This approach also builds on previous research exploring how frailty in older adults, characterized by decreased mobility and difficulty with daily activities, may contribute to reduced social engagement, increased loneliness, and further functional decline [7].

Our findings also align with existing evidence showing that both the presence and severity of global MBI symptoms are associated with frailty. This underscores the importance of assessing both cognitive and behavioral impairments when identifying individuals at risk of dementia [2]. However, given the relatively small sample size of frail individuals in this study, these findings should be interpreted with caution. Nonetheless, our results support prior research linking neuropsychiatric symptoms, such as psychosis, not only to an elevated risk of dementia but also frailty, which is often associated with accelerated cognitive decline [45]. To strengthen these observations, future research should consider analyzing larger cohorts with detailed assessments of domain-specific MBI symptoms and frailty. Such studies could yield more robust evidence, further elucidating the relationship between specific MBI domains and frailty and reinforcing their potential as targets for early intervention in individuals at risk of dementia and functional decline.

Our findings are consistent with those of a previous study that used data from the Korean Frailty and Aging Cohort Study, which found that less frequent contact with acquaintances was associated with a higher prevalence of frailty [46]. Similar patterns have been observed worldwide. For instance, a 1-year prospective cohort study in Japan demonstrated that greater social isolation, measured using the Lubben Social Network Scale, increased the risk of developing prefrail status [47]. Among Italian community-dwelling older adults, a significant relationship was found between frailty and higher levels of social isolation, measured using the Friendship Scale, and frailty [48]. Although the precise mechanisms through which social connections influence frailty are not yet fully understood, growing epidemiological evidence suggests that social interactions can affect health outcomes through psychosocial, behavioral, and biological pathways. A lack of social interactions, for instance, can amplify the sympathetic nervous system and the hypothalamus-pituitary-adrenal axis, thus leading to a chronic mild inflammatory state, which is characteristic of frailty syndrome [49-51]. Limited social interactions can also increase stress-related hormones such as glucocorticoids, impacting immune functions and potentially affecting the biological aspects of frailty [52]. Furthermore, frequent social interactions can motivate frail older adults to engage in cognitively and physically stimulating activities, providing daily support and health management resources [52,53].

Overall, our findings underscore the importance of social interaction correlates to frailty among older adults. This issue is particularly relevant in East Asian societies, where traditional family structures and intergenerational support are highly valued [54]. However, the rapid demographic shift toward an aging population and declining family size in developed East Asian countries, such as South Korea, Japan, and China, poses challenges to maintaining adequate social contact for older adults [54,55]. This phenomenon may increase the risk of frailty in older adults who lack sufficient social engagement due to changing family dynamics [56]. Furthermore, in developed countries, where health care systems are more established, promoting social functioning could be a cost-effective, nonpharmacological intervention to reduce frailty and its associated health care burden [57,58]. These findings suggest that public health policies aimed at enhancing social interactions, particularly for those at risk of cognitive decline, could reduce frailty and promote healthy aging in both East Asian and developed countries more broadly.

Our study did not establish a significant correlation between daily loneliness and frailty, in contrast to previous research. This discrepancy may be attributed to 2 primary reasons. First, our method of measuring loneliness was based on a single direct question covering a 2-week period. While we aimed to reflect on the participants’ subjective perceptions of their social interactions, a more in-depth, long-term study may be necessary to capture the full complexity of loneliness. A systematic review and meta-analysis of the relationship between loneliness and physical frailty in community-dwelling older adults indicated that baseline loneliness could predict worsening frailty, although longitudinal evidence is limited [59]. Therefore, future studies should focus on the cumulative effects of comprehensive potential factors prevalent in older adults, such as decreased physical activity, poor sleep quality, and malnutrition [59-61]. Additionally, these studies should examine these factors within the context of social vulnerability and frailty, while controlling for the effects of social networks [61].

Second, unlike the frequency of social interactions, loneliness is a subjective feeling, and perceptions of loneliness can vary significantly among individuals [7]. Typically, loneliness is seen as a negative response to the gap between actual and desired relationships [62]. However, the threshold for feeling lonely often depends on sociocultural context or personal standards [63,64]. Therefore, using more nuanced, context-specific questionnaires could lead to a deeper understanding of loneliness [7]. Nonetheless, given that our study participants were older adults at risk for dementia, using numerous detailed questionnaires might not be the most feasible approach. An alternative method could involve analyzing patterns among participants who provide similar responses to brief, directional questions, and then prospectively studying their perceptions of loneliness in response to specific events in a real-time setting. This approach could yield valuable insights into subjective experiences of loneliness in this population.

### Comparison With Prior Work

A key strength of our study lies in its detailed exploration of the relationship between social interaction and frailty status among older adults with SCD or MCI. By categorizing frailty into robust, prefrail, and frail groups, our findings build upon and extend previous research, indicating that older adults with higher levels of daily social interaction are less likely to exhibit frailty, even after accounting for cognitive and behavioral symptoms. While these findings align with prior research suggesting the protective effects of social interaction on health, our study adds nuance by examining these relationships specifically within older adults at risk of cognitive decline.

Our results support existing evidence that frail older adults exhibit lower social interaction frequency and higher loneliness levels compared with their robust and prefrail counterparts. These findings underscore the importance of social interaction as a critical behavioral dimension for health outcomes in older adults at risk of both cognitive and functional decline. While causation cannot be established, our findings suggest that fostering social connections may mitigate frailty risks and promote better health outcomes.

What distinguishes our study is its use of real-time data collection methods, which allowed us to capture day-to-day variations in social functioning with greater accuracy [40]. This approach offers a more granular understanding of how social interaction relates to frailty and cognitive status, building on prior retrospective studies. By applying real-time assessment within the context of frailty and cognitive decline, this study strengthens the evidence base for social interaction as a modifiable factor and highlights the need for future research to explore causative pathways.

Although the immediate practical application of these findings in clinical settings may be limited, this study contributes to the growing understanding of the relationship between social function, frailty, and cognitive decline. It highlights the potential for improving social engagement interventions that may reduce physical challenges targeted to older adults with SCD or MCI. Future research should focus on identifying the specific types, patterns, and contexts of social interaction that are most effective in reducing frailty and promoting health. Longitudinal and interventional studies will be crucial in establishing causation and designing tailored interventions to enhance social functioning in these at-risk populations.

### Limitations

This study had several limitations. First, the sample size of 101 participants, particularly the small sample size of the frail group, may limit the generalizability of the findings. A larger and more diverse sample could yield more robust results and allow for detailed analyses across different subgroups. Second, the cross-sectional design of the study enabled the identification of associations but did not establish causation. Prospective longitudinal studies are necessary to determine the directionality of the relationships among frailty, MBI symptoms, and social functioning. Additionally, Alzheimer disease pathology, hormonal changes, and chronic inflammation are potential mechanistic links between frailty and cognitive decline. While this study focuses on social interaction, physiological dimensions such as physical activity, metabolic health, and nutritional status were not assessed to minimize the participants’ measurement burden during the 2-week survey period. The primary aim of our study was to demonstrate how capturing temporal patterns in social functioning could inform daily life modifications to support health. Future research should integrate complementary physiological measures to enhance understanding and inform more comprehensive intervention strategies in longitudinal or experimental studies to better understand the directionality of these relationships. For example, studies using matching designs over extended timespans would be essential. Third, this study relied on self-reported data for loneliness levels and the tools used to measure daily social interaction frequency and loneliness levels, which may be susceptible to social desirability bias, which could influence how participants report their feelings and circumstances. Future analyses could incorporate digital devices, such as actigraphy, to log social interaction frequency and patterns, thereby integrating supplementary objective measures. Additionally, qualitative aspects of loneliness, such as its depth and contextual nuances, may require more comprehensive assessment methodologies. Tools like daily diaries or note-taking functions are advisable for future EMA approaches to capture more detailed information on the types and quality of social interactions. Moreover, framing questions with both positive and negative perspectives could mitigate response patterns where participants consistently answer in one direction, enhancing the robustness of findings in larger studies. Fourth, the use of a self-reported frailty assessment via the FPQ may introduce recall bias or inaccuracies in participants’ self-perceptions. While the FPQ is a validated and practical screening tool for community-dwelling older adults, it may lack the granularity of more detailed clinical assessments. Future studies could consider integrating tools such as the Clinical Frailty Scale to capture a broader spectrum of frailty characteristics and enhance precision in frailty classification. Finally, while our study provided step-by-step guidance and a helpdesk for participants to navigate the EMA app, applying the EMA approach via mobile devices to older adults less familiar with mobile technology requires more comprehensive and detailed guidance. Ensuring that participants can use the mobile app independently is an essential step for successfully replicating this study methodology.

### Conclusions

Our study suggests that daily social interaction is associated with frailty status among older adults with SCD or MCI, irrespective of the presence or severity of MBI symptoms. This finding builds on previous research, highlighting the positive potential of improving social interaction to reduce frailty risk among older adults with cognitive decline. To deepen understanding and enhance timely intervention strategies, future studies should explore the qualitative aspects of social interactions and frailty in greater depth. While the immediate clinical application of these findings may be limited, the use of real-time assessment in social functioning offers a promising path for further investigation. Integrating real-time social data with objective measures could provide a more personalized and comprehensive approach to care.

## Appendix

Multimedia Appendix 1 The timing of the four daily alarms.

Multimedia Appendix 2 First step to respond to the application.

Multimedia Appendix 3 Second step to respond to the application.

Multimedia Appendix 4 Distribution of mean maximum daily social interaction scores by frailty status, based on multiple daily scores for each participant. The black histogram represents robust participants, the grey histogram represents prefrail group, and the blue histogram represents frail group. Participants recorded the number of face-to-face meetings, phone calls, or video calls lasting more than five minutes using a five-point Likert scale (0 for “no contact,” 1 for “once,” 2 for “two times,” 3 for “three times,” and 4 for “four or more times”).

Multimedia Appendix 5 Distribution of mean maximum daily loneliness levels by frailty status, based on multiple daily scores for each participant. The black histogram represents robust participants, the grey histogram represents prefrail group, and the red histogram represents frail group. Participants assessed loneliness using a five-point Likert scale (0 = “very lonely” to 4 = “not lonely at all”), with higher scores indicating lower levels of loneliness.

## Abbreviations

EMA: ecological momentary assessment
FPQ: frailty phenotype questionnaire
K-IADL: Korean Instrumental Activities of Daily Living scale
MBI: mild behavioral impairment
MBI-C: Mild Behavioral Impairment Checklist
MCI: mild cognitive impairment
RRR: relative risk ratio
SCD: subjective cognitive decline

## Data Availability

The datasets used or analyzed in this study are available from the first author upon reasonable request.
