# Supplementary figures and images for: Assessing Social Interaction and Loneliness and Their Association With Frailty Among Older Adults With Subjective Cognitive Decline or Mild Cognitive Impairment: Ecological Momentary Assessment Approach
Source: JMIR Mhealth Uhealth. 2025 Apr 22;13:e64853. doi: 10.2196/64853 (PMC12056436; doi:10.2196/64853)

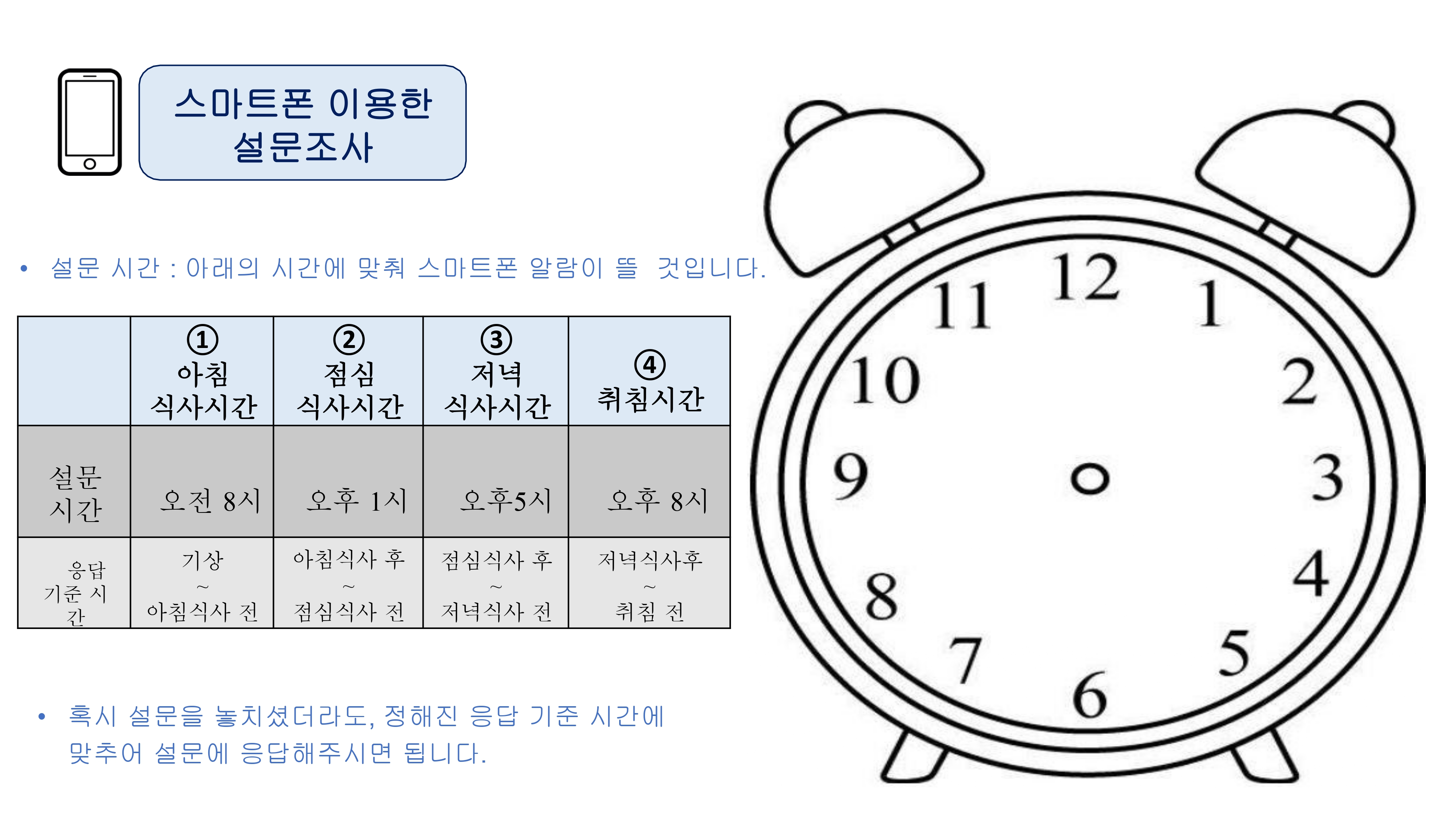

Supplement: Multimedia Appendix 1 [file mhealth_v13i1e64853_app1.png]

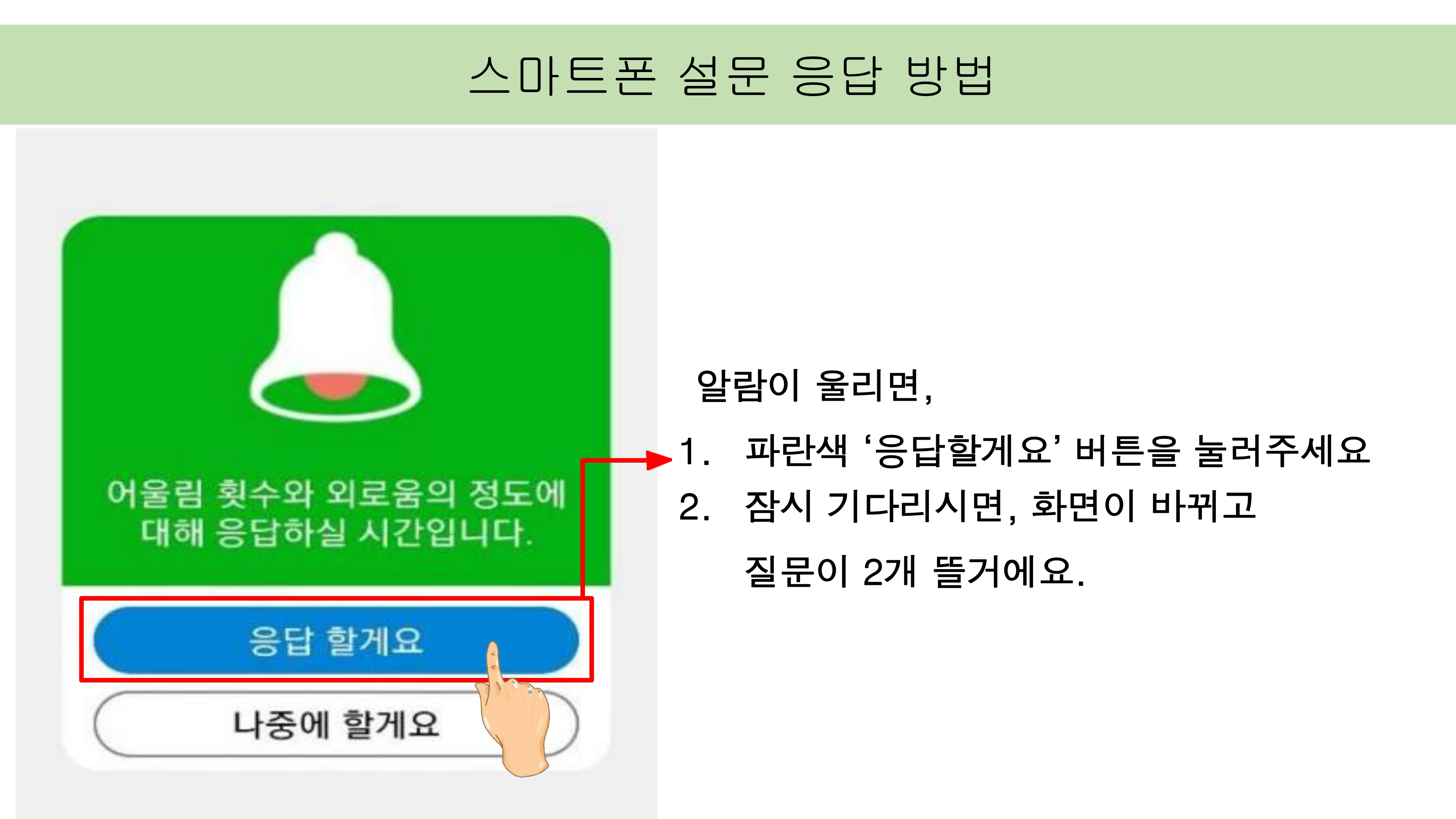

Supplement: Multimedia Appendix 2 [file mhealth_v13i1e64853_app2.png]

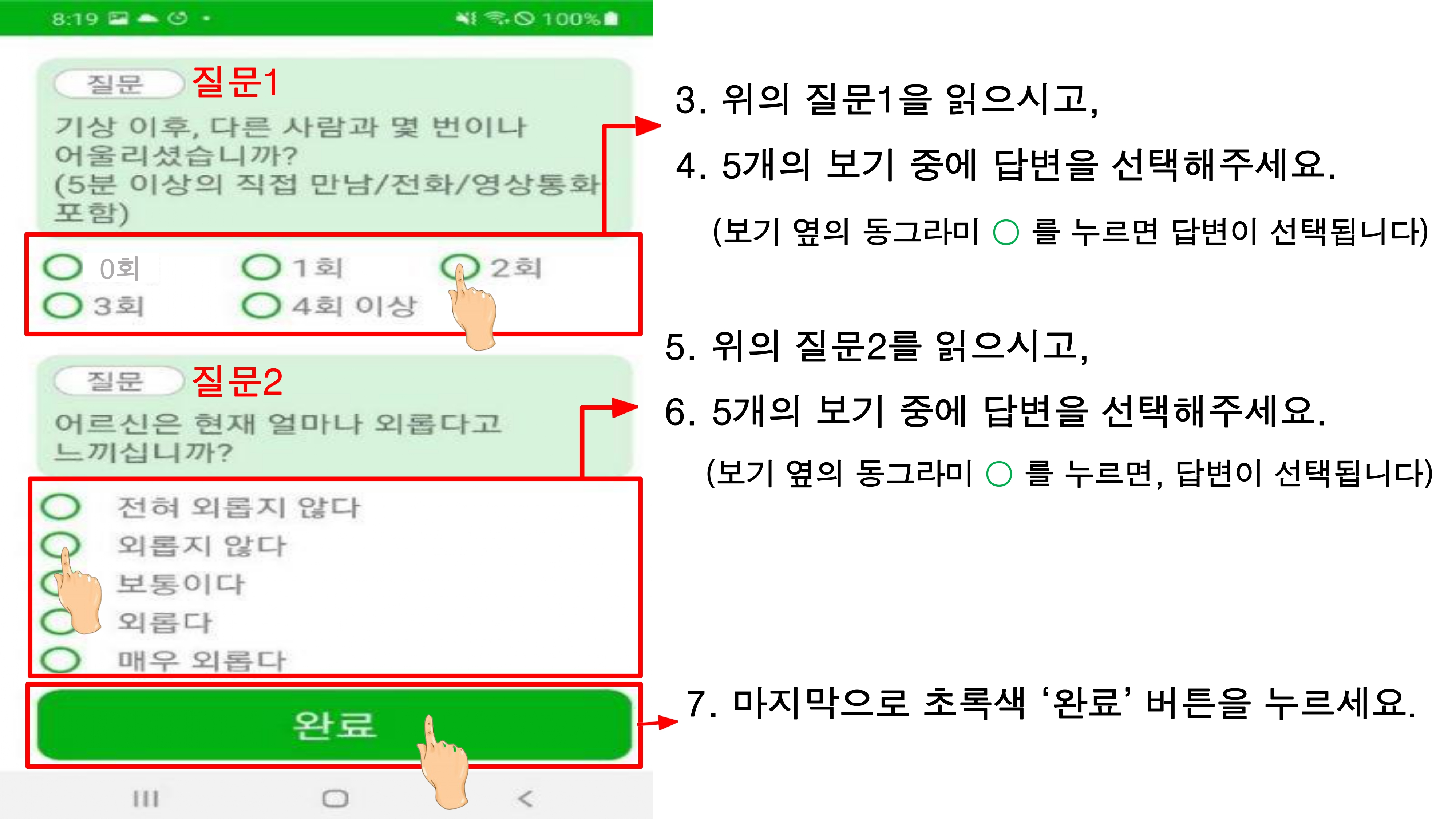

Supplement: Multimedia Appendix 3 [file mhealth_v13i1e64853_app3.png]

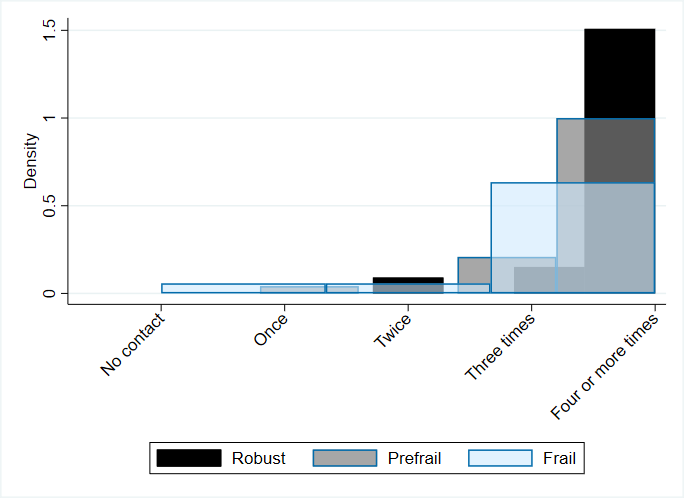

Supplement: Multimedia Appendix 4 [file mhealth_v13i1e64853_app4.png]

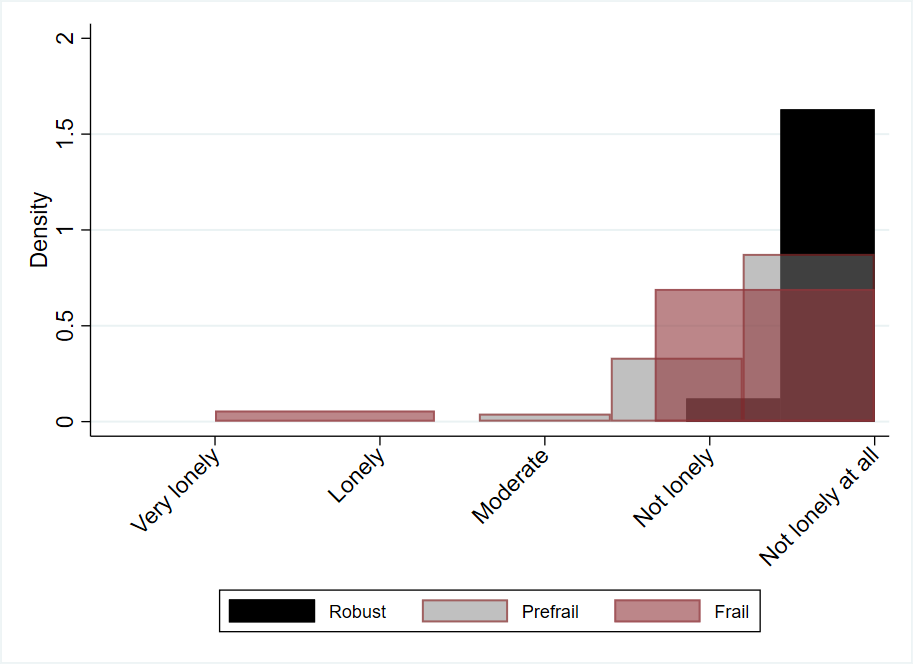

Supplement: Multimedia Appendix 5 [file mhealth_v13i1e64853_app5.png]
